# Co‐administration of Inulin and Iron Fortificants improves Iron Deficiency Biomarkers in Female Sprague Dawley Rats

**DOI:** 10.1002/fsn3.2337

**Published:** 2022-05-07

**Authors:** Abdul Momin Rizwan Ahmad, Umar Farooq, Mariam Anees, Riffat Aysha Anis, Summer Rashid, Waqas Ahmed

**Affiliations:** ^1^ Department of Nutrition & Dietetics National University of Medical Sciences (NUMS) Rawalpindi Pakistan; ^2^ Department of Diet and Nutritional Sciences IBADAT International University Islamabad Pakistan; ^3^ Department of Biochemistry Quaid‐i‐Azam University Lahore Pakistan; ^4^ Department of Food and Nutrition Minhaj University Lahore Pakistan; ^5^ Department of Food Science & Human Nutrition University of Veterinary & Animal Sciences Lahore Pakistan

**Keywords:** Inulin, Iron Deficiency, Iron Fortificants, Micronutrient deficiencies, Serum Ferritin, Serum Iron

## Abstract

Micronutrient deficiencies affect approximately 2 billion people worldwide and iron deficiency anemia is one of them. The instant research was an attempt to determine the efficacy of co‐administration of two iron fortificants (NaFeEDTA and FeSO_4_) and inulin (a prebiotic) on serum iron, ferritin, transferrin, and total iron‐binding capacity in iron‐deficient female Sprague Dawley rats. For this research, rats were divided into ten groups, (two control and eight treatment groups). Treatment groups were made iron deficient by feeding them with triapine, an iron binder for two weeks. All treatment groups were fed with inulin at two different dosage levels along with iron fortificants. The study results showed that serum ferritin and serum iron levels significantly improved from initiation to termination of study. Also, mean values of total iron‐binding capacity and serum transferrin showed a steady decline over a period of three months indicating that iron stores were being improved. It was concluded that co‐administration of inulin and iron fortificants helped improve iron deficiency biomarkers in female Sprague Dawley rats.

## Graphical abstract

The instant research was an attempt to determine the efficacy of co‐administration of two iron fortificants (NaFeEDTA and FeSO4) and Inulin (a prebiotic) on serum iron, ferritin, transferrin and total iron binding capacity in iron deficient female Sprague Dawley rats. The study results showed that serum ferritin and serum iron levels significantly improved from initiation to termination of study. Also, mean values of total iron binding capacity and serum transferrin showed a steady decline over a period of three months indicating that iron stores were being improved. It was concluded that co‐administration of Inulin and iron fortificants helped improve iron deficiency biomarkers in female Sprague Dawley rats.

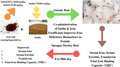

## 1 INTRODUCTION

Hidden hunger refers to a lack of minerals and vitamins and occurs when the quality of food being consumed by an individual is not good and does not fulfill the customized nutritional requirements of that particular individual (Gödecke et al., 2018). It is one of the most important public health issues worldwide and despite several interventions, the prevalence of this particular problem has been on a rise lately. Hidden hunger affects all age groups, but most commonly affected age groups include women of childbearing age (15‐49 years), under 5 children, and infants (Addis Alene & Mohamed Dohe, 2014).

Major contributors to micronutrient deficiencies include poor mineral bioavailability, lack of dietary diversity, enhanced physiological requirements, and high incidence of illness and disease. Poverty has been long recognized as one of the fundamental root causes of undernutrition. This is exactly why the majority of developing and underdeveloped countries have got the highest disease burden in terms of micronutrient deficiencies (Ahmad et al., 2020). The interesting fact though is that MNDs not only exist in underdeveloped and developed countries, but they are also found in specific population cohorts in high income countries (Bailey et al., 2015).

Anemia is simply referred to as the decreased oxygen‐carrying capacity of erythrocytes to tissues. The World Health Organization's cutoff values for anemia vary with sex, age, and status of pregnancy. Adult women who are nonpregnant are considered anemic when their hemoglobin shows a concentration of less than 12 g/dL, while pregnant women are diagnosed as anemic when their hemoglobin levels show a concentration of less than 11 g/dL (Alem et al., 2013).

A prebiotic is defined as a nonviable food substance component (insoluble fiber), which exhibits selective fermentation and moves to the colon (Hutkins et al., 2016). For foods which fall under the definition of prebiotics, they ought not to be absorbed or hydrolyzed in the upper part of the gastrointestinal tract, should have the ability to exhibit a positive influence on the gut microflora, should pose some beneficial effects to host health and should be a selective substrate for at least some of the colonic bacteria including Firmicutes, Actinobacteria, and Proteobacteria (Khramtsov et al., 2017).

Several research studies have shown that prebiotics help in the absorption of iron in both animal and human models. In addition, they are also known to exhibit positive effects on other bone‐related minerals, such as magnesium, calcium, and zinc, and thereby help to improve the Bone Mineral Content (BMC) (Laparra et al., 2014). It has also been suggested specifically that inulin tends to exhibit iron absorption increasing capabilities. This particular characteristic exhibited by both of these prebiotics can be exploited to treat iron deficiency anemia in humans which is one of the biggest public health issues worldwide (Tako et al., 2014).

Prebiotics such as and inulin and lactulose have been shown to increase serum iron biomarkers and other hematological indices including hemoglobin, hematocrit, and mean corpuscular volume, as concluded by the results of a study conducted by Masanetz *et al*. in 2011 (Masanetz et al., 2011).

To determine the effects of prebiotic supplementation on iron absorption in anemic rats, a study was carried out in which inulin and oligofructose were used as prebiotics. The results of the study revealed that inulin was able to enhance the levels of divalent metal transporter 1 (DMT1) in the cecum, which is a protein responsible for transfer of ferrous iron in humans and animals. The results also revealed that inulin elevated the levels of Duodenal cytochrome B (Dcyt B) in the colon, which is an enzyme that acts as a catalyst in the reduction of ferric (Fe^3+^) to ferrous (Fe^2+^) form in the process of iron absorption, both in humans and animals. The study concluded that inulin and oligofructose as prebiotics may have positive effects on the proteins, which regulate the absorption of iron in animals and humans (Marciano et al., 2015).

Another study was carried out by Petry *et al*. in 2012 to evaluate the effect of inulin on absorption of iron and bifidobacteria and short‐chain fatty acids among anemic women. The results of the study, however, showed that there was not much significant difference in the iron status of women who were supplemented with inulin. It was though evident from the study that inulin did decrease fecal pH (Petry et al., 2012).

## 2 MATERIALS & METHODS

The objective of the current study was to determine the effects of co‐administration of inulin and iron fortificants on various biomarkers associated with iron deficiency including serum iron, serum ferritin, transferrin, and total iron‐binding capacity.

The prior approval of the study was taken from the ERC (Ethical Review Committee) of the University of Veterinary & Animal Sciences, Lahore vide Letter No. DR/996 on 25 September 2018. Also, the experiment was performed in accordance with all the relevant institutional guidelines for the care and use of laboratory animals.

The instant research was conducted at the Animal House, National Institute of Health (NIH), Islamabad. For this study, n = 70 young female Sprague Dawley rats, who were 6 to 8 weeks of age, were obtained from NIH, Islamabad. All the required raw materials and chemicals needed for the instant research were bought from well‐reputed international companies.

Eight different types of iron fortificants and inulin‐based fortified feeds were manually prepared for the purpose of this study. While iron fortificants were added to the feed of rats, inulin was dissolved in water to be fed to rats on daily basis. Commercially available inulin and iron fortificants were used for the current study. As per the labels of the commercial varieties, inulin was 98.6% pure while both iron fortificants, that is, NaFeEDTA and FeSO_4_ had a purity of >99%.

For calculation of dosage of inulin in mg/kg body weight of rats, HED (Human Equivalent Dose) formula was used; (Shin et al., 2010).

$$\text{HED}\;\text{(mg}/\text{kg)}=\text{Animal}\;\text{Dose}\;\text{in}\;\text{mg}/\text{kg}\times \frac{{\left(\text{Animal}\;\text{Weight}\;\text{in}\;\text{kgs}\right)}^{0.33}}{\left(\text{Human}\;\text{Weight}\;\text{in}\;\text{kgs}\right)}$$

Two iron fortificants were used in the trials namely ferrous sulfate (FeSO_4_) and ferric sodium ethylenediaminetetraacetate (NaFeEDTA). Doses of these compounds were 15 and 30 mg/kg of diet for FeSO_4_ and 10 and 20 mg/kg of diet for NaFeEDTA. These two doses were selected on the basis of iron fortification guidelines of the World Health Organization (Dary & Hurrell, 2006).

The rats were divided into ten groups (seven rats in each group), The groups were made as described in Table 1.

**TABLE 1:** Treatment plan for female sprague dawley rats

GROUP	DIET PLAN	HUMAN EQUIVALENT DOSE (HED) ‐ FOR INULIN
G_+_	Basal Diet +Water/No Added Fortificant/No Inulin ‐ Fed to Healthy Rats	‐
G_‐_	Basal Diet +Water/No Added Fortificant/No Inulin ‐ Fed to Iron‐Deficient Rats	‐
G_1_	Basal Diet +722 mg/kg Inulin +10 mg/kg NaFeEDTA	100 mg/kg =6 grams
G_2_	Basal Diet +722 mg/kg Inulin +20 mg/kg NaFeEDTA	100 mg/kg =6 grams
G_3_	Basal Diet +963 mg/kg Inulin +10 mg/kg NaFeEDTA	133 mg/kg =8 grams
G_4_	Basal Diet +963 mg/kg Inulin +20 mg/kg NaFeEDTA	133 mg/kg =8 grams
G_5_	Basal Diet +722 mg/kg Inulin +15 mg/kg FeSO_4_	100 mg/kg =6 grams
G_6_	Basal Diet +722 mg/kg Inulin +30 mg/kg FeSO_4_	100 mg/kg =6 grams
G_7_	Basal Diet +963 mg/kg Inulin +15 mg/kg FeSO_4_	133 mg/kg =8 grams
G_8_	Basal Diet +963 mg/kg Inulin +30 mg/kg FeSO_4_	133 mg/kg =8 grams

Rats were initially acclimatized by providing them with a standard feed for a period of one week before the start of experiment. Anemia was then induced in rats by feeding them with triapine, an iron binder. Once anemia was induced, evident by lethargy in rats and their excessive hair fall, baseline values were attained which also served as a cross‐check to determine that anemia had been actually induced in rats. After that, rats were fed with iron fortificants and inulin‐based feed daily for a period of 90 days. Basal diet of rats consisted of 82% wheat flour, 10% corn oil, 4% casein, 3% minerals (including 35 mg/kg of iron), and 1% vitamins mixture. Iron was added to this basal diet in treatment groups in the form of either NaFeEDTA or FeSO_4_ from commercially available sources. Rats were weighed at the start of each week so as to adjust the dosage of inulin for the subsequent week. Housing of rats was done in stainless steel cages whereby a temperature of 23±2ºC and a humidity of 55 ± 5% were maintained throughout the experimental period. In addition, it was ensured that a light–dark cycle of 12 hours was also maintained.

Blood samples of rats were collected in fasting state at 0 day, 30th day, 60th day, and 90th day from tail vein. For determination of serum iron (Wojciak et al., 2013), serum ferritin (Kazuaki et al., 2011), and serum transferrin (Al‐Buhairan & Oluboyede, 2001), their respective protocols were used. Total iron‐binding capacity (TIBC) was calculated on the basis of serum transferrin using the formula:

Total Iron‐Binding Capacity =Transferrin x 24.

Statistical analysis of the obtained data was done to determine the level of significance. For this purpose, SPSS version 23.0 was employed. Analysis of variance under factorial design was used for purpose of statistical analysis and differences were considered significant at P‐value <0.05 (Johnson & Bhattacharyya, 2019).

## 3 RESULTS

### 3.1 Serum iron

The results presented in Table 2 clearly show that an increase in serum iron levels was recorded when anemic rats were fed with iron fortificants and inulin fortified diet. Maximum increase could be observed in groups G_4_ (90.28 ± 11.39µg/dL), G_3_ (90.02 ± 7.07µg/dL), G_8_ (89.39 ± 7.78 µg/dL) and G_5_ (87.03 ± 6.54µg/dL), respectively. It can be observed that three out of four of these groups had Inulin dosage of 963 mg/kg body weight of rats which indicates that this dose was more effective compared to the lower dose of 722 mg/kg body weight dose when combined with either of two iron fortificants.

Among the groups, maximum increase in serum iron levels along the progression of study intervals was witnessed in group G_4_, G_8_, G_3_ and G_6_, respectively. For group G_4_, the value of serum iron at 0 day was recorded to be as 77.88 ± 1.81µg/dL which increased to 103.81 ± 2.02 µg/dL at 90th day. For group G8, this value rose from 79.45 ± 0.73µg/dL at baseline to 96.67 ± 1.47µg/dL at 90th day. Serum iron levels reported in groups G3 and G6 ranged from 80.74 ± 3.15µg/dL and 78.08 ± 1.63µg/dL to 97.51 ± 2.18µg/dL and 93.08 ± 1.53µg/dL, respectively. As expected, no improvement in serum iron levels was seen in group G−, whereby the levels decreased from 77.21 ± 2.47µg/dL at 0 day to 62.78 ± 2.07µg/dL at 90th day since this group was not fed with any iron fortificants or Inulin.

**TABLE 2:** Serum Iron Levels (µg/dL) among Anemic Female Rats from Baseline up to 90 days

Treatments/ Groups	Days				Means
	0	30	60	90	
G_+_	83.80 ± 1.36^a^	82.62 ± 1.63^bc^	82.25 ± 1.78^c^	83.02 ± 1.64^e^	82.92 ± 0.66^abc^
G_‐_	77.21 ± 2.47^b^	70.23 ± 3.07^c^	65.73 ± 2.83^d^	62.78 ± 2.07^f^	68.98 ± 6.28^d^
G_1_	74.15 ± 2.48^b^	77.72 ± 3.43^d^	83.44 ± 1.90^c^	85.92 ± 1.52^e^	80.3 ± 5.35^c^
G_2_	78.14 ± 3.08^b^	83.22 ± 2.27^cd^	82.95 ± 1.77^c^	86.49 ± 1.47^e^	82.7 ± 3.44^bc^
G_3_	80.74 ± 3.15^b^	89.20 ± 3.35^a^	92.65 ± 2.48^ab^	97.51 ± 2.18^b^	90.02 ± 7.07^a^
G_4_	77.88 ± 1.81^b^	84.64 ± 2.80^bcd^	94.82 ± 3.86^a^	103.81 ± 2.02^a^	90.28 ± 11.39^a^
G_5_	80.43 ± 3.96^b^	82.54 ± 2.22^bcd^	91.36 ± 1.69^ab^	93.8 ± 1.30^cd^	87.03 ± 6.54^ab^
G_6_	78.08 ± 1.63^b^	82.19 ± 1.89^cd^	90.48 ± 1.57^b^	93.08 ± 1.53^d^	85.95 ± 7.01^abc^
G_7_	78.75 ± 1.48^b^	82.45 ± 1.90^cd^	85.06 ± 2.65^c^	87.13 ± 3.08^e^	83.34 ± 3.61^bc^
G_8_	79.45 ± 0.73^b^	87.11 ± 1.89^ab^	94.36 ± 1.98^ab^	96.67 ± 1.47^bc^	89.39 ± 7.78^a^

Note

Means carrying the same letters in a column are not significantly different. Where, G_+_, Unfortified feed (healthy control); G_‐_, Unfortified feed (anemic control); G_1_, Basal Diet +722 mg/kg Inulin +10 mg/kg NaFeEDTA; G_2_, Basal Diet +722 mg/kg Inulin +20 mg/kg NaFeEDTA; G_3_, Basal Diet +963 mg/kg Inulin +10 mg/kg NaFeEDTA; G_4_, Basal Diet +963 mg/kg Inulin +20 mg/kg NaFeEDTA; G_5_, Basal Diet +722 mg/kg Inulin +15 mg/kg FeSO_4_; G_6_, Basal Diet +722 mg/kg Inulin +30 mg/kg FeSO_4_; G_7_, Basal Diet +963 mg/kg Inulin +15 mg/kg FeSO_4_; G_8_, Basal Diet +963 mg/kg Inulin +30 mg/kg FeSO_4_.

### 3.2 Serum ferritin

When rats were fed with fortified diet, an increase in serum ferritin levels was seen as is evident from Table 3. Serum ferritin levels increased by maximum in group G_4_ (53.00 ± 25.29ng/mL) and this group was followed by groups G_8_ (48.63 ± 21.10ng/mL), G_3_ (45.07 ± 19.56ng/mL), G_2_ (43.61 ± 20.35ng/mL) and G_1_ (41.70 ± 20.03ng/mL). Four out of these five groups were fed with NaFeEDTA combined with Inulin which indicates that in terms of increase in serum ferritin levels, NaFeEDTA performed better compared to FeSO_4_.

During the rat trials, a considerable increase in levels of serum ferritin could be clearly observed. As far as the groups were concerned, a similar noticeable increase in serum ferritin levels was recorded whereby group G4 performed the best whose values ranged from 19.51 ± 0.70ng/mL at the start to 77.81 ± 1.14ng/mL at the end of study. This was followed by groups G_8_, G_3_, G_2_ and G_1_, whose values from initiation to termination were recorded as 19.78 ± 0.96ng/mL to 68.33 ± 1.26ng/mL, 19.39 ± 0.94ng/mL to 64.74 ± 0.86ng/mL, 19.46 ± 0.75ng/mL to 63.37 ± 2.39ng/mL and 19.49 ± 0.74ng/mL to 63.28 ± 2.45ng/mL, respectively.

**TABLE 3:** Serum Ferritin Levels (ng/mL) among Anemic Female Rats from Baseline up to 90 days

Treatments/ Groups	Days				Means
	0	30	60	90	
G_+_	24.24 ± 1.75^a^	23.64 ± 1.43^d^	23.42 ± 1.16^f^	24.8 ± 1.22^f^	24.02 ± 0.62^cd^
G_‐_	19.87 ± 0.83^b^	16.78 ± 0.30^e^	15.33 ± 1.44^g^	14.02 ± 0.95^g^	16.50 ± 2.51^d^
G_1_	19.49 ± 0.74^bc^	31.2 ± 5.81^c^	57.38 ± 2.20^bc^	63.28 ± 2.45^d^	42.83 ± 20.90^ab^
G_2_	19.46 ± 0.75^bc^	34.43 ± 1.06^c^	57.18 ± 1.29^c^	63.37 ± 2.39^d^	43.61 ± 20.35^ab^
G_3_	19.39 ± 0.94^bc^	41.64 ± 1.18^b^	54.53 ± 1.26^d^	64.74 ± 0.86^c^	45.07 ± 19.56^ab^
G_4_	19.51 ± 0.70^bc^	48.9 ± 1.28^a^	65.81 ± 1.58^a^	77.81 ± 1.14^a^	53.00 ± 25.29^a^
G_5_	19.66 ± 0.78 ^bc^	33.2 ± 1.42^c^	42.04 ± 1.28^e^	46.71 ± 1.11^e^	35.40 ± 11.90^bc^
G_6_	18.91 ± 0.74^bc^	33.24 ± 1.68^c^	40.16 ± 2.09^e^	46.53 ± 1.93^e^	34.71 ± 11.85^bc^
G_7_	18.91 ± 0.81^bc^	33.08 ± 1.40^c^	41.19 ± 1.76^e^	47.35 ± 1.24^e^	35.13 ± 12.29^bc^
G_8_	19.78 ± 0.96^bc^	47.13 ± 1.56^a^	59.28 ± 4.19^b^	68.33 ± 1.26^b^	48.63 ± 21.10^a^

Note

Means carrying the same letters in a column are not significantly different. Where, G_+_, Unfortified feed (healthy control); G_‐_, Unfortified feed (anemic control); G_1_, Basal Diet +722 mg/kg Inulin +10 mg/kg NaFeEDTA; G_2_, Basal Diet +722 mg/kg Inulin +20 mg/kg NaFeEDTA; G_3_, Basal Diet +963 mg/kg Inulin +10 mg/kg NaFeEDTA; G_4_, Basal Diet +963 mg/kg Inulin +20 mg/kg NaFeEDTA; G_5_, Basal Diet +722 mg/kg Inulin +15 mg/kg FeSO_4_; G_6_, Basal Diet +722 mg/kg Inulin +30 mg/kg FeSO_4_; G_7_, Basal Diet +963 mg/kg Inulin +15 mg/kg FeSO_4_; G_8_, Basal Diet +963 mg/kg Inulin +30 mg/kg FeSO_4_.

### 3.3 Serum transferrin

In our study, maximum value for serum transferrin levels was recorded in group G_‐_ (21.06 ± 0.12mg/dL), followed by G_1_ (20.48 ± 0.48mg/dL), G_7_ (20.39 ± 0.42mg/dL), G_2_ (20.37 ± 0.51mg/dL), G_3_ (20.06 ± 0.92mg/dL), G_5_ (20.01 ± 0.70mg/dL), G_8_ (19.93 ± 0.90mg/dL), G_4_ (19.86 ± 1.05mg/dL), G_6_ (19.78 ± 0.82mg/dL), and G_+_ (19.50 ± 0.05mg/dL). Among the groups, group G_4_ performed the best whose value for serum transferrin varied from 20.99 ± 0.20mg/dL at the start of study to 18.52 ± 0.17mg/dL at the termination. This was followed by group G_8_, group G_3,_ and group G_6_. For group G_8_, the value ranged from 20.97 ± 0.20mg/dL at 0 day to 18.81 ± 0.33mg/dL at 90th day while for group G_3_, the level of serum ferritin varied from 20.96 ± 0.20mg/dL at the start of study to 18.90 ± 0.16mg/dL at the termination. For group G_6_, the value was recorded to be 20.97 ± 0.17mg/dL at 0 day and it decreased to 19.12 ± 0.06mg/dL at 90th day. A similar trend of considerable decrease in serum transferrin levels was also observed in terms of study intervals(Table 4).

**TABLE 4:** Serum Transferrin Levels (mg/dL) among Anemic Female Rats from Baseline up to 90 days

Treatments/ Groups	Days				Means
	0	30	60	90	
G_+_	19.52 ± 0.23^b^	19.53 ± 0.20^e^	19.52 ± 0.11^de^	19.43 ± 0.16^d^	19.50 ± 0.05^f^
G_‐_	20.93 ± 0.08^a^	21.00 ± 0.09^a^	21.10 ± 0.10^a^	21.21 ± 0.06^a^	21.06 ± 0.12^a^
G_1_	21.01 ± 0.12^a^	20.66 ± 0.11^b^	20.38 ± 0.14^b^	19.88 ± 0.14^bc^	20.48 ± 0.48^b^
G_2_	20.87 ± 0.17^a^	20.68 ± 0.15^b^	20.22 ± 0.23^b^	19.74 ± 0.20^c^	20.37 ± 0.51^bcd^
G_3_	20.96 ± 0.20^a^	20.61 ± 0.17^b^	19.78 ± 0.15^c^	18.90 ± 0.16^e^	20.06 ± 0.92^bcde^
G_4_	20.99 ± 0.20^a^	20.27 ± 0.22^c^	19.66 ± 0.17^cd^	18.52 ± 0.17^f^	19.86 ± 1.05^def^
G_5_	21.05 ± 0.21^a^	19.79 ± 0.15^d^	19.72 ± 0.13^cd^	19.49 ± 0.09^d^	20.01 ± 0.70^bcdef^
G_6_	20.97 ± 0.17^a^	19.68 ± 0.12^de^	19.36 ± 0.07^e^	19.12 ± 0.06^e^	19.78 ± 0.82^ef^
G_7_	21.00 ± 0.25^a^	20.30 ± 0.12^c^	20.21 ± 0.12^b^	20.06 ± 0.05^b^	20.39 ± 0.42^bc^
G_8_	20.97 ± 0.20^a^	20.19 ± 0.09^c^	19.78 ± 0.24^c^	18.81 ± 0.33^e^	19.93 ± 0.90^cdef^

Note

Means carrying the same letters in a column are not significantly different. Where, G_+_, Unfortified feed (healthy control); G_‐_, Unfortified feed (anemic control); G_1_, Basal Diet +722 mg/kg Inulin +10 mg/kg NaFeEDTA; G_2_, Basal Diet +722 mg/kg Inulin +20 mg/kg NaFeEDTA; G_3_, Basal Diet +963 mg/kg Inulin +10 mg/kg NaFeEDTA; G_4_, Basal Diet +963 mg/kg Inulin +20 mg/kg NaFeEDTA; G_5_, Basal Diet +722 mg/kg Inulin +15 mg/kg FeSO_4_; G_6_, Basal Diet +722 mg/kg Inulin +30 mg/kg FeSO_4_; G_7_, Basal Diet +963 mg/kg Inulin +15 mg/kg FeSO_4_; G_8_, Basal Diet +963 mg/kg Inulin +30 mg/kg FeSO_4_.

### 3.4 Total iron‐binding capacity

TIBC determines the total amount of iron which transferrin can carry. Transferrin is actually a protein which binds iron to be transported throughout the body. Total iron‐binding capacity is often employed as an indicator of iron deficiency anemia. When an individual develops anemia, TIBC levels tend to rise which is an indication that iron stores are decreased in the body. (Whitney & Rolfes, 2018).

Means regarding total iron‐binding capacity levels shown in Table 5 clearly depict that maximum value was recorded in group G_‐_ (505.50 ± 2.90µg/dL) which was followed by group G_1_ (491.54 ± 11.49µg/dL), G_7_ (489.39 ± 10.08µg/dL), G_2_ (489.11 ± 12.17µg/dL), and group G_3_ (481.48 ± 22.09µg/dL). The subsequent maximum values of TIBC were observed in groups G_5_ (480.30 ± 16.90µg/dL), G_8_ (478.50 ± 21.60µg/dL), G_4_ (476.64 ± 25.13µg/dL), G_6_ (474.79 ± 19.79µg/dL), and G_+_ (468.02 ± 1.08µg/dL). Among the treatment groups, maximum decline in TIBC levels occurred in group G_4_, G_8_, G_3,_ and G_6_. In group G_4_, the levels decreased steadily from 503.79 ± 4.81µg/dL at 0 day to 444.43 ± 3.98µg/dL at 90th day while in group G_8_, these levels declined from 503.32 ± 4.80µg/dL at initiation to 451.44 ± 7.96µg/dL at termination. For group G_3_ and G_6_, serum TIBC levels decreased from 502.99 ± 4.81µg/dL and 503.31 ± 4.19µg/dL to 453.57 ± 3.72µg/dL and 458.87 ± 1.33µg/dL at 0 and 90th day, respectively. Likewise, a steadily declining trend of TIBC levels was also observed study intervals wise.

**TABLE 5:** Total Iron‐Binding Capacity Levels (µg/dL) among Anemic Female Rats from Baseline up to 90 days

Treatments/ Groups	Days				Means
	0	30	60	90	
G_+_	468.48 ± 5.44^a^	468.73 ± 4.75^e^	468.47 ± 2.71^de^	466.41 ± 3.76^d^	468.02 ± 1.08^f^
G_‐_	502.37 ± 1.96^b^	504.11 ± 2.24^a^	506.48 ± 2.39^a^	509.04 ± 1.48^a^	505.50 ± 2.90^a^
G_1_	504.26 ± 2.95^b^	495.72 ± 2.70^b^	489.18 ± 3.33^b^	477.00 ± 3.39^bc^	491.54 ± 11.49^b^
G_2_	500.94 ± 4.06^b^	496.40 ± 3.53^b^	485.39 ± 5.46^b^	473.71 ± 4.72^c^	489.11 ± 12.17^bcd^
G_3_	502.99 ± 4.81^b^	494.74 ± 4.19^b^	474.63 ± 3.71^c^	453.57 ± 3.72^e^	481.48 ± 22.09^bcde^
G_4_	503.79 ± 4.81^b^	486.49 ± 5.27^c^	471.88 ± 4.12^cd^	444.43 ± 3.98^f^	476.64 ± 25.13^def^
G_5_	505.21 ± 4.97^b^	475.07 ± 3.67^d^	473.28 ± 3.18^cd^	467.65 ± 2.19^d^	480.30 ± 16.90^bcdef^
G_6_	503.31 ± 4.19^b^	472.31 ± 2.81^de^	464.67 ± 1.61^e^	458.87 ± 1.33^e^	474.79 ± 19.79^ef^
G_7_	504.09 ± 6.11^b^	487.10 ± 2.91^c^	484.95 ± 2.89^b^	481.42 ± 1.10^b^	489.39 ± 10.08^bc^
G_8_	503.32 ± 4.80^b^	484.56 ± 2.17^c^	474.71 ± 5.78^c^	451.44 ± 7.96^e^	478.50 ± 21.60^cdef^

Note

Means carrying the same letters in a column are not significantly different. Where, G_+_, Unfortified feed (healthy control); G_‐_, Unfortified feed (anemic control); G_1_, Basal Diet +722 mg/kg Inulin +10 mg/kg NaFeEDTA; G_2_, Basal Diet +722 mg/kg Inulin +20 mg/kg NaFeEDTA; G_3_, Basal Diet +963 mg/kg Inulin +10 mg/kg NaFeEDTA; G_4_, Basal Diet +963 mg/kg Inulin +20 mg/kg NaFeEDTA; G_5_, Basal Diet +722 mg/kg Inulin +15 mg/kg FeSO_4_; G_6_, Basal Diet +722 mg/kg Inulin +30 mg/kg FeSO_4_; G_7_, Basal Diet +963 mg/kg Inulin +15 mg/kg FeSO_4_; G_8_, Basal Diet +963 mg/kg Inulin +30 mg/kg FeSO_4_.

## 4 DISCUSSION

In the past, numerous researches studies have been conducted which have shown that addition of prebiotics to the diet of iron‐deficient animal and human models increased iron absorption (Wang, 2017). Moreover, addition of prebiotics to diet has not only resulted in improved gut health but has also significantly enhanced bone mineral content, as per the results of various studies (Scholz‐Ahrens et al., 2001).

A study was conducted to determine if addition of different prebiotics such as oligosaccharides, lactulose, and inulin to the diet could affect iron status of anemic rats or not. The study results revealed that absorption of iron significantly improved in treatment groups compared to the control group. Moreover, the study showed that when prebiotics were added to the diet, short‐chain fatty acids (SCFAs) were produced in abundance, indicating increased fermentation of prebiotics in the colon, which eventually led to enhanced iron absorption (Zhang, 2017).

The researchers conducted another study recently in the year 2021 to determine the effects of prebiotics on iron status of anemic rats. This study concluded that prebiotics were slightly able to improve the iron status of anemic rats (Zhang, Yung, & KongYeung, 2021). In another study in 2020, the researchers tried to determine the effects of iron fortified yogurt (supplemented with short chain fatty acids produced by prebiotics) on various blood parameters of anemic rats. The results of this study showed that not only the hemoglobin and hematocrit levels of anemic rats were improved after consumption of fortified yogurt, but also the Red blood cell count was significantly improved (Mohammed, Dyab, Kheadr, & Dabour, 2021).

Among prebiotics, inulin has been specifically found to be extremely effective in terms of iron absorption in both animals and humans (Shoaib et al., 2016). In a study conducted by peers in 2016, it was demonstrated that inulin could significantly enhance the levels of iron absorption in male piglets (Lepczyński et al., 2016). In another instance, the researchers established that women fed with a prebiotic mixture had improved iron absorption at the termination of the study, compared to the control group (Weinborn et al., 2017).

In a study conducted by Rubi and Rohman, iron deficiency anemia was induced in female Wistar rats and was divided into different groups based on provision of either FeSO_4_ (6, 12, and 24 mg/kg) or NaFeEDTA (6, 12, and 24 mg/kg). Serum iron levels of rats when assessed revealed that maximum serum iron levels were gained by group fed with FeSO_4_ @ 24 mg/kg whereby the value was 139.36 ± 1.58μg/dL. The group fed with NaFeEDTA @ 24 mg/kg, however, showed mean serum iron levels of 134.10 ± 2.73μg/dL (Sudargo et al., 2015). Our study results are also in accordance with this study as we have also reported that serum iron levels of anemic rats fed with either FeSO_4_ or NaFeEDTA were increased steadily over a period of 90 days.

In another study, anemic elderly in Boston, USA, were given iron‐fortified grain products on daily basis for a period of 6 to 8 months. On the other hand, the control group only got grain products without fortification of iron. At the end of study, hemoglobin and serum iron levels of both the groups were compared and it was seen that the group which received fortified grain products had increased levels of hemoglobin as well as serum iron. The researchers concluded that iron fortification of grain products helps anemic elderly regain their normal hemoglobin as well as serum iron levels (Gershoff et al., 1977). Our study results are exactly in accordance with this particular study as we have also concluded that iron fortification tends to improve serum iron levels in anemic subjects.

Ferritin is the storage form of iron in an individual, and serum ferritin stores tend to diminish in the initial stage of iron deficiency. This is why serum ferritin levels are of utmost importance in terms of diagnosis of iron deficiency anemia. Although recommended often because of cost‐effectiveness and ease, hemoglobin is not a reliable indicator of iron deficiency anemia as hemoglobin levels drop fairly late in case of anemia (Whitney & Rolfes, 2018).

Rubi and Rohman have previously reported an increase in serum ferritin levels in anemic female Wistar rats when they were fed with NaFeEDTA and FeSO_4_‐based feed. They reported that serum ferritin levels increased in both the groups fed with either NaFeEDTA or FeSO_4_‐fortified diet. However, mean ferritin levels of both these groups were not significantly different from each other . Our study results are in harmony with this particular research as in our case too, mean serum ferritin levels of all the treatment groups of anemic rats have significantly increased from the initiation of study to termination at 90th day.

Earlier in year 2003, a research was conducted in Vietnam whereby an evaluation of efficacy of fish sauce fortified with iron was done as fish sauce is one of the staple foods of the region. It was a randomized controlled trial in which 152 anemic women were served with fish sauce containing 10‐mg iron in the form of NaFeEDTA for a period of 6 months. At the time of initiation of study, baseline values of hemoglobin and serum ferritin were obtained and the same data were collected at 90th and 180th day. After a period of 6 months, it was observed that mean value for hemoglobin was 116.30 ± 8.7g/L in the fortified group, compared to control group in which the mean value was recorded to be 107.60 ± 11.0g/L (P‐value <0.0001). Similarly, mean value for serum ferritin levels was found out to be 30.90 (95% CI: 23.4, 40.6) μg/L in the fortified group compared to 14.6 (11.3, 19.0) μg/L in the control group. (P‐value =0.0002) (Thuy et al., 2003). Our study results are also in accordance with this research study as we have also stated that serum ferritin levels in anemic subjects steadily increased from 0 day to 90th day when anemic subjects were given iron and inulin‐fortified diet.

Serum transferrin is considered to be a relatively novel indicator of iron deficiency anemia as it has not been in use for diagnosis of iron deficiency anemia for long. It has been shown to increase when an individual develops iron deficiency or when the process of erythropoiesis gets increased (Asobayire et al., 2001). A few studies have been recently conducted which have indicated that serum transferrin is a reliable and useful indicator for diagnosis of iron deficiency anemia specifically in individuals suffering from malaria or other infections. Serum transferrin has also been specifically shown to respond to iron supplementation in iron‐deficient human subjects (Zhu & Haas, 1998).

A Vietnamese research in 2003 has also concluded that serum transferrin levels in anemic women steadily declined over a period of 6 months, when they were fed with iron‐fortified fish sauce (Thuy et al., 2003). Our study results have also shown similar results as there was a steady decline in serum transferrin levels of anemic rats over a period of 90 days. The steady decline in serum transferrin levels over the study duration indicated that iron stores were being regained by anemic women. A decline in serum transferrin is favorable for anemic subjects because it indicates that anemia is being rectified and lesser iron is needed subsequently by the body (Cable et al., 2016).

Our study results are also in accordance with the results of study conducted in 2020 by researchers who reported that addition of galacto‐oligosaccharides helped enhance absorption of iron in female rat models (Ahmad et al., 2019).

Similarly, another study closely related to our study results conducted in 2014 indicated that when iron deficient rats were administered prebiotics, their plasma Hepcidin levels were reduced, resulting in increased absorption of iron (Laparra et al., 2014).

## 5 CONCLUSION

Our study concluded that fortification of feed with inulin and NaFeEDTA or FeSO_4_ helped improve biomarkers associated with iron deficiency in female Sprague Dawley rats. This indicated that inulin had some iron absorption enhancing capabilities, although its exact mechanism is still not known. This particular kind of fortification could help resolve one of the biggest public health problems, being faced by the worldwide community in the present day and age.

## CONFLICT OF INTEREST

The corresponding author on the behalf of all authors declares that there is no conflict of interest in submitting the findings of instant research in the journal.

## ETHICAL APPROVAL

The prior approval of the study was taken from the ERC (Ethical Review Committee) of the University of Veterinary & Animal Sciences, Lahore vide Letter No. DR/996 on 25 September 2018. Also, the experiment was performed in accordance with all the relevant institutional guidelines for the care and use of laboratory animals.

## Data Availability

Data available on request from the authors
