#  Erratum

**DOI:** 10.1002/fsn3.3086

**Published:** 2022-10-09

Erratum to “Co‐administration of Inulin and Iron Fortificants Improves Iron Deficiency Biomarkers in Female Sprague Dawley Rats”

Rizwan Ahmad, AM, Farooq, U, Anees, M, et al. Co‐administration of Inulin and Iron Fortificants Improves Iron Deficiency Biomarkers in Female Sprague Dawley Rats. *Food Sci Nutr*. 2022; 10: 2141–2148. https://doi.org/10.1002/fsn3.2337

In the originally published version of the article, authors Sanaullah Iqbal and Mavra Javed were erroneously omitted. The authors and their affiliations have now been corrected in the article. Also, in affiliation 3, the state name was changed from “Lahore” to “Islamabad.” Below is the updated list of authors and corresponding affiliations:

Abdul Momin Rizwan Ahmad^1^ | Umar Farooq^2^ | Mariam Anees^3^ | Riffat Aysha Anis^2^ | Sanaullah Iqbal^4^ | Summer Rashid^5^ | Mavra Javed^4^ | Waqas Ahmed^4^

^1^Department of Nutrition & Dietetics, National University of Medical Sciences (NUMS), Rawalpindi, Pakistan

^2^Department of Diet and Nutritional Sciences, IBADAT International University, Islamabad, Pakistan

^3^Department of Biochemistry, Quaid‐i‐ Azam University, Islamabad, Pakistan

^4^Department of Food Science & Human Nutrition, University of Veterinary & Animal Sciences, Lahore, Pakistan

^5^Department of Food and Nutrition, Minhaj University, Lahore, Pakistan

